# Combined prognostic value of the cancer stem cell markers CD47 and CD133 in esophageal squamous cell carcinoma

**DOI:** 10.1002/cam4.1894

**Published:** 2019-02-11

**Authors:** Jian‐Hua Wang, Shu‐Ting Huang, Lan Zhang, Zi‐Gang Liu, Rong‐Xin Liang, Sen‐Wei Jiang, Yi‐Nan Jiang, Xing‐Juan Yu, Yu‐Chuan Jiang, Xi‐Zhao Li, Pei‐Fen Zhang, Zhe‐Sheng Wen, Min Zheng

**Affiliations:** ^1^ Department of Chest Second People’s Hospital of Guangdong Province Guangzhou China; ^2^ Department of Gynecology Sun Yat‐Sen University Cancer Center Guangzhou China; ^3^ State Key Laboratory of Oncology in South China Guangzhou China; ^4^ Collaborative Innovation Center for Cancer Medicine Sun Yat‐Sen University Cancer Center Guangzhou China; ^5^ Department of Chest Sun Yat‐Sen University Cancer Center Guangzhou China

**Keywords:** CD133, CD47, CSCs, ESCC, prognosis, therapeutic

## Abstract

**Background:**

Treatments based on the inhibition of pivotal signals of cancer stem cells (CSCs) are on a promising track. Recent studies have shown that targeting CSCs with broader immune‐based therapeutic methods, for example, the anti‐CD47 treatment, may serve as a more potent strategy for eliminating these intractable cells. We aimed to explore the prognostic effects of CD47/CD133 and the potential therapeutic significance of CD47 in esophageal squamous cell carcinoma (ESCC).

**Methods:**

Immunohistochemistry was employed to identify the characteristics of CD47 and CD133 in 26 pairs of tumor tissues and adjacent non‐tumor tissues and 136 ESCC tissues. Kaplan‐Meier analysis and Cox proportional hazards models were built for estimating the prognostic values of CD47 and CD133 expression and their combined stemness index. Sphere formation assays were undertaken to explore the effects of CD47 inhibition on primary human ESCC CSCs.

**Results:**

Results conclude that CD47 and CD133 expression is increased in tumor tissues as compared to adjacent non‐tumor tissues. A positive correlation between CD47/CD133 expression and differentiation was found in 136 ESCC patients. Survival analysis indicated that patients with high CD47 or CD133 expression exhibited poor overall survival and progression‐free survival (PFS). The combination of high CD47 and CD133 expression was a reliable independent prognostic factor for both OS (HR = 1.940, 95% CI = 1.399‐2.690, *P* < 0.0001) and progression‐free survival (HR = 1.883, 95% CI = 1.384‐2.562, *P* < 0.0001). Notably, CD47+ CD133+ ESCC cells were observed to possess the characteristics of CSCs, and anti‐CD47 treatment veritably eliminated the CSCs pool.

**Conclusions:**

The stemness index determined by the expression of CD47 and CD133 is a promising prognostic predictor, and CD47 is a potential therapeutic target for CSCs in ESCC patients.

## 1 INTRODUCTION

Esophageal cancer, ranking as the sixth most common cause of cancer‐related deaths, is termed a major global cancer burden.^1^ In China, esophageal cancer accounted for 477 900 incident cases and 375 000 deaths in 2015,^2^ about 90% of which fall under the category of esophageal squamous cell carcinoma (ESCC).^3^ Despite improvements in diagnostics and therapeutics, the prognosis of ESCC remains poor, with a median 5‐year survival of <25%,^4^ thus lending to an urgent need to resolve the molecular mechanism of ESCC in order to develop biomarkers and targeted therapies.

CD47, also known as integrin‐associated protein (IAP), is a transmembrane protein of the immunoglobulin superfamily. Cancer cells expressing CD47 transmit a “don't eat me” signal upon interacting with signal regulatory protein α on the surface of macrophages to deter phagocytosis.^5^ Previous studies have shown that CD47 is highly expressed on solid tumors such as in ovarian, breast, colon, bladder, glioblastoma, hepatocellular carcinoma and prostate cancer,^6^ and correlated with poorer prognoses in several cancer types including ESCC.^6^, ^7^, ^8^ Furthermore, anti‐CD47 antibody or CD47 blockade treatments have been demonstrated to enhance macrophage phagocytosis, reduce tumor burden, and increase patient survival in various tumor xenograft models.^9^

Increasing evidence denotes a minority population of cells within tumors, termed cancer stem cells (CSCs), as critical determinants in cancer recurrence, metastasis, and therapy resistance.^10^ CSCs have been identified by several cell surface markers, such as CD133,^11^ CD44,^12^ and CD90.^13^ However, neutralizing antibodies against these markers have proven insufficient in eradicating CSCs in preclinical studies, and identification of new therapeutic targets against CSCs is necessary. Preferentially, CD47 was proven to be expressed in CSCs of pancreatic,^14^ liver,^15^ and lung cancers^16^ in these years. Knockdown of CD47 suppressed certain stem‐like properties of cancer cells, such as self‐renewal and chemoresistance,^14^, ^15^, ^16^, ^17^ suggesting that targeting CD47 could not only active the phagocytosis of macrophages, but also be refined into a potent supplement in treatment against CSCs. Up until now, no study dedicated to clarifying the association between CD47 expression and CSCs characteristics in ESCC exists.

In this study, we demonstrated that increased CD47 protein levels in ESCC clinical samples correlated with the expression of CD133, which is the most robust CSCs marker. A combination of CD47 and CD133 improved prognostic stratification of ESCC survival. Furthermore, inhibition of CD47 by a neutralizing antibody suppressed the self‐renewal function in CD133^+^ ESCC cells, thereby making the case for CD47 being a promising therapeutic target for ESCC CSCs.

## 2 MATERIALS AND METHODS

### 2.1 Patients and surgical specimens

We randomly enrolled 136 patients diagnosed with ESCC who underwent esophagectomy at the Sun Yat‐sen University Cancer Center between 2002 and 2009, and additionally collected paired tumor and adjacent tumor tissues from 26 patients. Clinicopathological parameters were obtained from medical records. None of the patients underwent anti‐cancer therapies before surgery, and no histologically confirmed serious complications or other malignant diseases had been reported. Tumor stages were determined according to the classification system of the Union for International Cancer Control (UICC), 7th Edition, while grading and histopathology subtyping for tumor differentiation was based on World Health Organization criteria. According to UICC, the ≥66% extent of invasion depth was defined as tumor infiltration exceeding the intrinsic muscularis. Data were censored at the last follow‐up for patients without recurrence or death. Overall survival (OS) was defined as the date of surgery to death or the last follow‐up. Progression‐free survival (PFS) was the interval between surgery and recurrence, the last observation for patients without recurrence, or death if no recurrence was observed.

### 2.2 Immunohistochemistry

Immunohistochemistry (IHC) was performed using protocols described in a previous study.^18^, ^19^ Formalin‐fixed 10 hours at room temperature,paraffin‐embedded tissues were cut into 4‐μm sections and then sequentially dried, dewaxed, and re‐hydrated with xylene and a decreasing ethanol series. The slides were soaked in 0.3% H_2_O_2_ for 10 minutes to block endogenous peroxidase activity and boiled in 10 mmol/L citrate buffer (pH6.0) for 10 minutes for antigen retrieval. The sections of ESCC samples were then incubated with primary antibodies against CD47 (1:500; R&D systems, AF4670, Minneapolis, MN, USA) or CD133 (1:200; Abnova, PAB12663, Taiwan, China) overnight at 4°C. These antibodies were diluted by dilution buffer (Zhongshanjinqiao, Beijing, China, ZLI‐9028) and replaced by PBS as a blank control and replaced by Sheep IgG Control (1:500; R&D, 5‐001‐A) and Rabbit IgG Isotype control (1:400; Cell Signaling Technology, 3900, Boston, MA, USA). After that the sections were rinsed by PBS for 10 minutes each to wash out unspecific signal. The EnVision System with diaminobenzidine (Dako Cytomation, K5007, Copenhagen, Denmark) was used to carry out secondary antibody staining at 37°C for 30 minutes and detect signal. Brown color indicated positive staining. All sections were visualized by microscope (Nikon Eclipse 80i; Tokyo, Japan) and evaluated in high‐power fields (400×). CD47 and CD133 expression in tumor cells was measured via *H*‐score method. A final score was obtained by computing the percentage of positive staining cells (0%‐100%) in each staining intensity category (0‐3^+^): (3 percentage of strong staining) + (2 × percentage of moderate staining) + (percentage of weak staining), giving an *H*‐score range of 0‐300. Staining with isotype antibody was used as negative control. The IHC sections were examined by two independent pathologists blind to each patient’s clinical information. If their results were inconsistent, we would ask the third pathologist to evaluate. The strong, moderate, and weak staining of CD47 and CD133 were shown in Figure S2.

### 2.3 Sphere formation and antibody block assay

The CSCs sorting were performed using the method described by Lonardo et al with minor modifications.^20^ Fresh ESCC tissues were cut into fine crumbles and digested 30 minutes in 37°C by 50 μg/mL collagenase. Cells were obtained after filtering the digestion products and adjusted to a concentration of 10^6^ cells/mL in sorting buffer (1× PBS). Spheres were formed via culturing 3 × 10^3^ primary ESCC cells, obtained from flow cytometer (Gallios, AV28109, California, USA) using CD133‐FITC (Miltenyi Biotec, 130104322, Cologne, Germany) and CD47‐PE (Biolegend, 323108, San Diego, CA) after staining (in 1× PBS 30 μL) at room temperature for 30 minutes. The media of serum‐free DMEM/F12 had to contain certain key component supplementing with B27 (1:50; Invitrogen, Carlsbad, California, USA), 20 ng/mL epidermal growth factor (EGF; 1:5000; R&D Systems), 20 ng/mL basic fibroblast growth factor (bFGF; 1:5000; R&D Systems) for a total of 7 days, allowing spheres to reach a size of >70 mm. For subsequent passaging, mature spheres, filter through 40‐μm cell strainers, were dissociated into single cells, and then recultured in media with anti‐CD47 (2 μg/mL; R&D systems, AF4670) or anti‐IgG control antibody (2 μg/mL; R&D, 5‐001‐A) for another 7 days.

### 2.4 Statistical analysis

All statistical analyses were performed by GraphPad Prism 6 (GraphPad Software; United States) and IBM SPSS Soft 21 (IBM Corporation; United States). Pearson’s *χ* ^2^ test or Fisher’s exact test was used to examine the relationship between CD47/CD133 expression and clinicopathological parameters as appropriate. The correlation between CD47 and CD133 staining was determined by Pearson correlation analysis. Survival curves were calculated using the Kaplan‐Meier method and compared via log‐rank test. Prognostic variables with effects on survival in univariate analysis were included in a multivariate Cox proportional hazard regression model. A threshold of *P* < 0.05 denoted statistical significance.

## 3 RESULTS

### 3.1 CD47/CD133 is expressed at higher levels in ESCC compared with adjacent non‐tumor tissues

**Figure 1:**
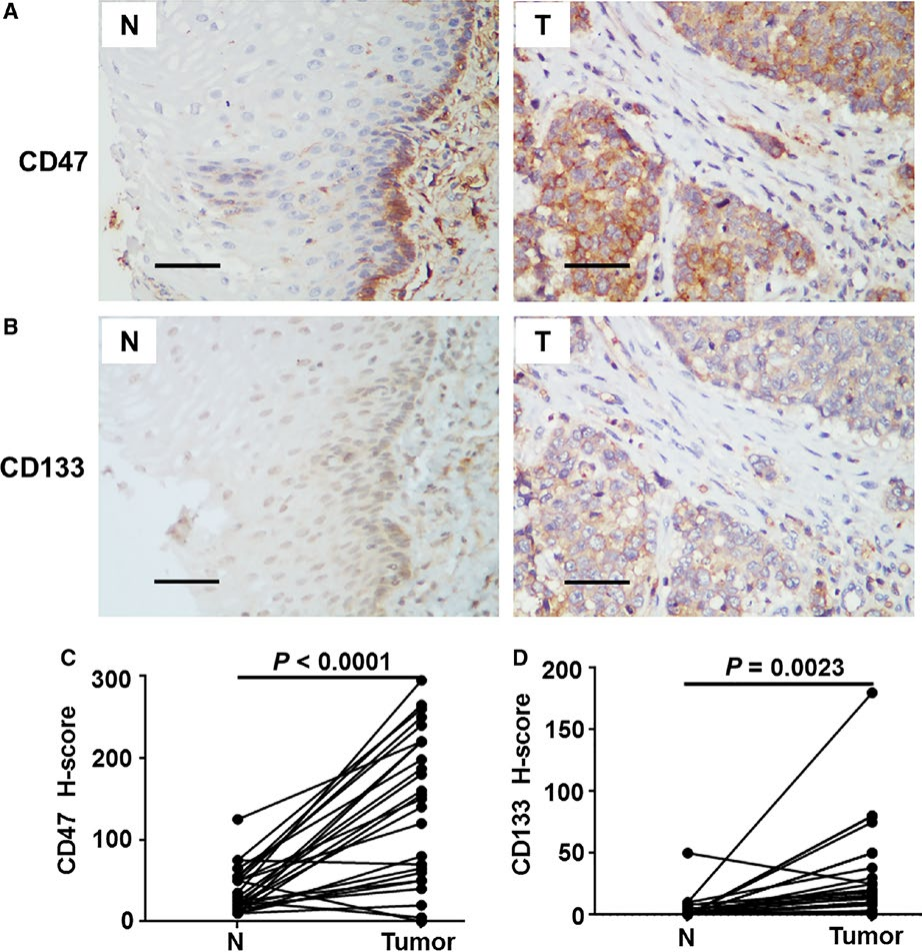
Characteristics of CD47 and CD133 expression in esophageal squamous cell carcinoma tissues. (A, B) Representative immunohistochemistry (IHC) images of CD47 (A) and CD133 (B) expression in 26 paired (n = 26) tumor (T) and adjacent non‐tumor tissues (N). The scale bar indicates 50 μm. (C, D) Statistical analyses of CD47 and CD133 expression in match paired tumor and adjacent non‐tumor tissues. The IHC *H*‐scores are shown as a symbol‐line plot from at least two independent pathologists. Differences were assessed with a paired two‐tailed *t* test

CD47 displayed clear membrane staining in 26 ESCC specimens through IHC staining, but barely any staining in adjacent esophagus cells with the exception of epidermal basal layer cells (Figure 1A), which are known for possessing a strong ability to divide. CD47 expression, quantified by the IHC *H*‐score, was significantly stronger in tumor tissues compared with the adjacent non‐tumor tissues (*P* < 0.0001; Figure 1C). Similarly, esophagus cells all stained negatively or minimally in adjacent noncancerous regions for CD133 (Figure 1B), whereas staining was often increased in the tumor (*P* = 0.011, Figure 1D).

### 3.2 CD47 and CD133 expression and their correlation with clinicopathological characteristics

We summarized the clinicopathological features of 136 ESCC patients in Table 1. The median follow‐up was 43 months (range from 1.6 to 130 months). During the follow‐up period, 75 patients (50.7%) died and 88 (59.5%) patients suffered from tumor progression, with the median OS and PFS being 44 and 32 months. Based on their CD47 and CD133 expression level, the patients were divided into groups following the cut‐off value (125 and 20, respectively) as inferred from the receiver operating characteristic (ROC) curve.

**Table 1:** Clinical characteristics of patients with ESCC

Clinical Variables	No. of patients	%
Gender		
Male	102	68.9
Female	46	31.1
Age (y)		
<60	87	58.8
≥60	61	41.2
Clinical stage		
I	3	2
II	82	55.4
III	62	41.9
IV	1	0.7
T classification		
T1	6	4.1
T2	29	19.6
T3	104	70.3
T4	9	6.1
N classification		
N0	75	50.7
N1	72	48.6
N2	1	0.7
M classification		
M0	147	99.3
M1	1	0.7
Depth of invasion		
<66%	43	29.1
≥66%	96	64.9
Location		
Upper	10	6.8
Middle	96	64.9
Lower	42	28.4
Differentiation		
Well	25	16.9
Moderate	74	50.0
Poor	47	31.8
Recurrence		
No	60	40.5
Yes	88	59.5
Vital status (at follow‐up)		
Alive	73	49.3
Death	75	50.7

**Figure 2:**
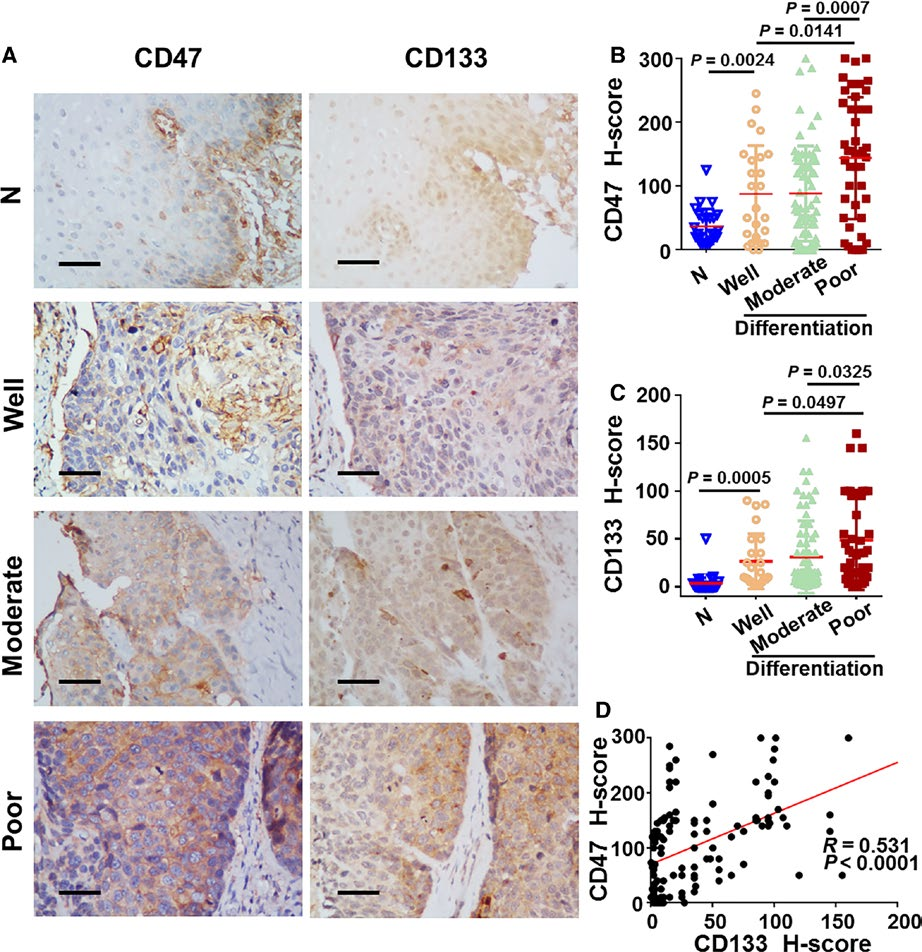
Association between CD47 and CD133 expression levels and tumor histological differentiation. A, Protein staining by IHC for CD47 and CD133 in 26 adjacent non‐tumor tissues and 136 tumors tissues. Scale bar = 50 μm. The mean CD47 (B) and CD133 (C) staining score was increased in poor (CD47 n = 44; CD133 n = 52) differentiation tumors compared with well (CD47 n = 25; CD133 n = 25) or moderate (CD47 n = 63; CD133 n = 59) differentiation tumors, and significantly higher than that in adjacent non‐tumor tissues regions. Data are expressed as mean ± SD (bars); (D) CD47 expression levels are positively correlated with CD133. The IHC *H*‐scores are examined by at least two independent pathologists. Differences were assessed with an unpaired two‐tailed *t* test. The IHC *H*‐scores are examined by at least two independent pathologists

We further analyzed the relationship between the patients’ CD47 and CD133 status and clinicopathological characteristics. Table 2 shows that expressions of CD47/CD133 and histological differentiation, recurrence and vital status were obviously correlated. Patients with poor differentiation tended toward a stronger expression of CD47 or CD133 (Figure 2A‐C). Furthermore, a positive correlation existed between CD47 and CD133 expression (*r* = 0.531, *P* < 0.0001, Figure 2D)

**Table 2:** Correlations between CD47/CD133 expression and clinicopathological features of patients with ESCC

Clinical variables	CD47 expression		*P* value	CD133 expression		*P* value
	Low (%)	High (%)		Low (%)	High (%)	
Gender						
Male	53 （70.7）	40 （70.2）	0.551	44 （60.3）	50 （80.6）	**0.008**
Female	22 （29.3）	17 （29.8）		29 （39.7）	12 （19.4）	
Age (y)						
<60	44（58.7）	34 （59.6）	0.526	45 （61.6）	32 （51.6）	0.159
≥60	31（41.3）	23 （40.4）		28 （38.4）	30 （48.4）	
Clinical stage						
I/II	45 （60.0）	30 （52.6）	0.252	39 （53.4）	35 （56.5）	0.429
III/IV	30 （40.0）	27 （47.4）		34 （46.6）	27 （43.5）	
T classification						
T1‐T2	18 （24.0）	13 （22.8）	0.521	15 （20.5）	15 （24.2）	0.381
T3‐T4	57 （76.0）	44 （77.2）		58 （79.5）	47 （75.8）	
N classification						
N0	42 （56.0）	25 （43.9）	0.114	37 （50.7）	31 （50.0）	0.537
N1‐2	33 （44.0）	32 （56.1）		36 （49.3）	31 （50.0）	
Depth of invasion						
<66%	23 （33.3）	16 （29.1）	0.379	23 （32.9	17 （29.8）	0.432
≥66%	46 （66.7）	39 （70.9）		47 （67.1）	40 （70.2）	
Location						
Upper	6 （8.0）	3 （5.3）	0.722	4 （5.5）	5 （8.1）	0.202
Middle	49 （65.3）	36 （63.2）		52 （71.2）	35 （56.5）	
Lower	20 （26.7）	18 （31.6）		17 （23.3）	22 （35.5）	
Differentiation						
Well/Moderate	61 （81.3）	32 （58.2）	0.004	55 （76.4）	36 （59.0）	**0.025**
Poor	14 （18.7）	23 （41.8）		17 （23.6）	25 （41.0）	
Recurrence						
No	40 （53.3）	14 （24.6）	0.001	36 （49.3）	19 （30.6）	**0.021**
Yes	35 （46.7）	43 （75.4）		37 （50.7）	43 （69.4）	
Vital status (at follow‐up)						
Alive	44 （58.7）	20 （35.1）	0.006	40 （54.8）	24 （38.7）	**0.045**
Death	31 （41.3）	37 （64.9）		33 （45.2）	38 （61.3）	

The Pearson’s chi‐squared test and Fisher’s exact test were used for analysis.

*P*‐values in bold indicates significance (*P* < 0.05).

### 3.3 High CD47 and CD133 expression in tumor cells predicts poor prognosis

**Figure 3:**
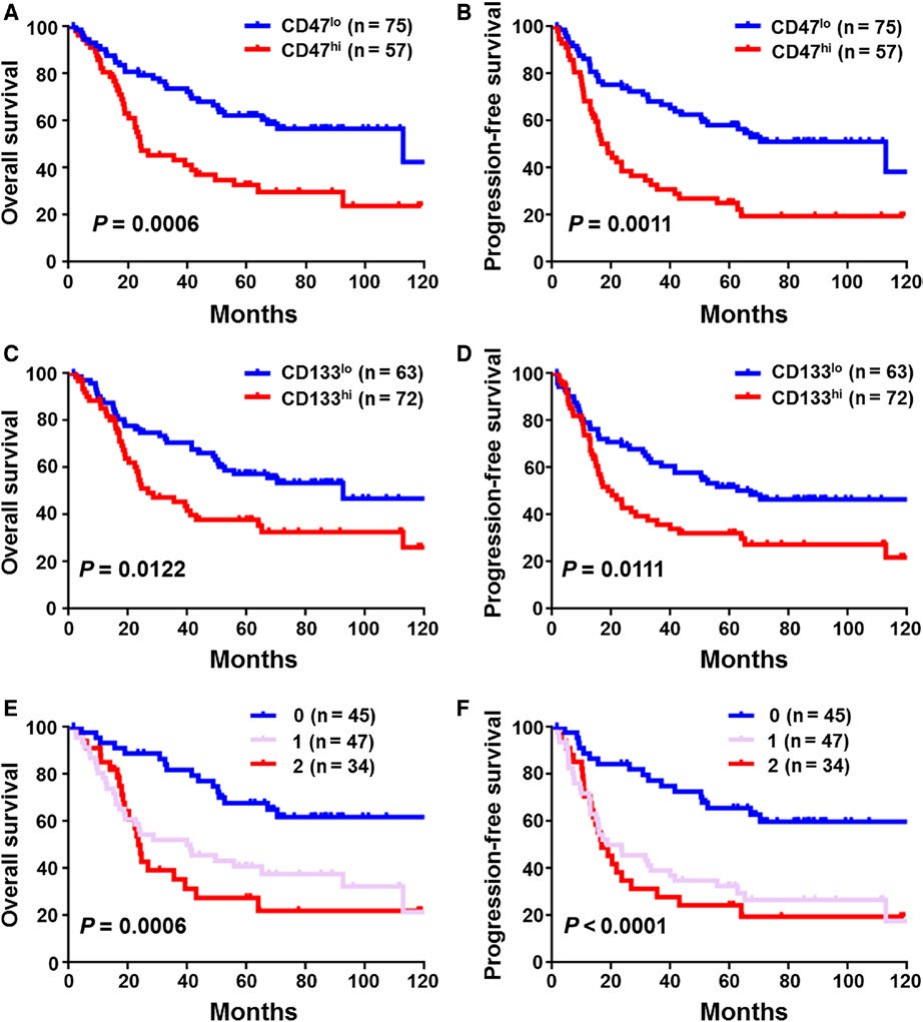
Cumulative survival curves of CD47, CD133, and the stemness index for esophageal squamous cell carcinoma patients. (A, B) Patients with higher CD47 (n = 57) expression have shorter overall survival (OS) and progression‐free survival (PFS) than whom in lower group (n = 75). (C, D) Compared with patients in low CD133 expression (n = 63), higher expression of CD133 (n = 72) is positively correlated with poor OS and PFS. (E, F) Higher stemness index (n = 34) predicts poor OS and PFS than stemness index “0” group (n = 45). The OS and RFS curves were generated by the Kaplan‐Meier method and analyzed using the log‐rank test

The relationship between the expression of CD47/CD133 and patients’ survival was investigated. Kaplan‐Meier survival curves and a log‐rank test were conducted, certifying that patients with high CD47 expression had shorter OS (*P* = 0.0006, Figure 3A) and PFS (*P* = 0.001, Figure 3B).Similarly, high CD133 indicated worse OS (*P* = 0.012, Figure 3C) and PFS (*P* = 0.011, Figure 3D) than patients with low expression. These results demonstrate that both CD47 and CD133 are effective predictors of prognosis in ESCC patients.

To assess whether CD47 or CD133 could be utilized as an independent indicator of OS/PFS, we performed a multivariate Cox proportional hazards analysis. As illustrated in Table 3, CD47/CD133 expression was associated with an increased risk of tumor progression in ESCC patients.

**Table 3:** Univariate and multivariate analysis of factors associated with survival and recurrence

Variable	Subset	Hazard ratio (95%)	*P* value
Overall survival			
Univariate analysis			
Gender	Male vs Female	0.783 (0.473‐1.298)	0.343
Age (y)	<60 vs ≥60	1.300 (0.826‐2.046)	0.257
Clinical stage	I‐II vs III‐IV	1.931 (1.225‐3.045)	**0.005**
T classification	T1‐T2 vs T3‐T4	1.916 (1.052‐3.491)	**0.034**
N classification	N0 vs N1‐2	1.919 (1.207‐3.051)	**0.006**
Depth of invasion (%)	<66 vs ≥66	2.064 (1.173‐3.630)	**0.012**
Location	Upper, Middle vs Lower	1.322 (0.876‐1.996)	0.184
Differentiation	Well, Moderate vs Poor	1.633 (1.024‐2.603)	**0.039**
CD47 expression	High vs Low	2.289 (1.412‐3.709)	**0.001**
CD133 expression	High vs Low	1.813 (1.131‐2.906)	**0.013**
Multivariate analysis			
N classification	N0 vs N1‐2	2.049 (1.227‐3.421)	**0.006**
Depth of invasion (%)	<66 vs ≥66	1.868 (1.037‐3.362)	**0.037**
CD47 expression	High vs Low	1.699 (1.013‐2.849)	**0.044**
CD133 expression	High vs Low	1.950 (1.153‐3.297)	**0.013**
Recurrence‐free survival			
Univariate analysis			
Gender	Male vs Female	0.877 (0.557‐1.381)	0.572
Age (y)	<60 vs ≥60	1.137 (0.748‐1.730)	0.547
Clinical stage	I‐II vs III‐IV	1.598 (1.049‐2.435)	**0.029**
T classification	T1‐T2 vs T3‐T4	1.495 (0.890‐2.514)	0.129
N classification	N0 vs N1‐2	1.834 (1.201‐2.802)	**0.005**
Depth of invasion (%)	<66 vs ≥66	1.910 (1.149‐3.174)	**0.013**
Location	Upper, Middle vs Lower	1.202 (0.831‐1.740)	0.329
Differentiation	Well, Moderate vs Poor	1.618 (1.047‐2.500)	**0.030**
CD47 expression	High vs Low	2.476 (1.573‐3.898)	<**0.0001**
CD133 expression	High vs Low	1.766 (1.132‐2.755)	**0.012**
Multivariate analysis			
N classification	N0 vs N1‐2	2.266 (1.390‐3.694)	**0.001**
CD47 expression	High vs Low	1.793 (1.096‐2.933)	**0.020**
CD133 expression	High vs Low	1.980 (1.203‐3.694)	**0.007**

*P*‐values in bold indicates significance (*P* < 0.05).

### 3.4 Prognostic significance of the stemness index in ESCC

CD47 and CD133 serve important roles in maintaining CSCs. Our findings indicate CD47 or CD133 expression presents a valuable independent factor in predicting the OS of ESCC. As such, we took a further step to combine CD47 and CD133 expression statistics into a stemness index to assess their effects in ESCC. Three groups were classified: Group 0, with low CD47 and low CD133; 1, low CD47 and high CD133 or high CD47 and low CD133; and 2, high CD47 and high CD133. Kaplan‐Meier analysis showed that patients in the 0 group exhibited the best OS *P* = 0.0006, Figure 3E and PFS *P* < 0.0001, Figure 3F compared with 1 and 2, while the stemness index for 2 was correlated with poor survival. Furthermore, a multivariate Cox analysis (Table 4) determined the stemness index as an independent prognostic factor for both OS and PFS in ESCC patients.

**Table 4:** Univariate and multivariate analysis of stemness status associated with survival and recurrence

Variable	Subset	Hazard ratio (95%)	*P* value
Overall survival			
Univariate analysis			
Gender	Male vs Female	0.783 (0.473‐1.298)	0.343
Age (y)	<60 vs ≥60	1.300 (0.826‐2.046)	0.257
Clinical stage	I‐II vs III‐IV	1.931 (1.225‐3.045)	**0.005**
T classification	T1‐T2 vs T3‐T4	1.916 (1.052‐3.491)	**0.034**
N classification	N0 vs N1‐2	1.919 (1.207‐3.051)	**0.006**
Depth of invasion (%)	<66 vs ≥66	2.064 (1.173‐3.630)	**0.012**
Location	Upper, Middle vs Lower	1.322 (0.876‐1.996)	0.184
Differentiation	Well, Moderate vs Poor	1.633 (1.024‐2.603)	**0.039**
Stemness status	0, 1 vs 2	1.774 (1.308‐2.407)	**<0.0001**
Multivariate analysis			
Clinical stage	I‐II vs III‐IV	2.080 (1.254‐3.453)	**0.005**
Stemness status	0, 1 vs 2	1.940 (1.399‐2.690)	**<0.0001**
Recurrence‐free survival			
Univariate analysis			
Gender	Male vs Female	0.877 (0.557‐1.381)	0.572
Age (y)	<60 vs ≥60	1.137 (0.748‐1.730)	0.547
Clinical stage	I‐II vs III‐IV	1.598 (1.049‐2.435)	**0.029**
T classification	T1‐T2 vs T3‐T4	1.495 (0.890‐2.514)	0.129
N classification	N0 vs N1‐2	1.834 (1.201‐2.802)	**0.005**
Depth of invasion (%)	<66 vs ≥66	1.910 (1.149‐3.174)	**0.013**
Location	Upper, Middle vs Lower	1.202 (0.831‐1.740)	0.329
Differentiation	Well, Moderate vs Poor	1.618 (1.047‐2.500)	**0.030**
Stemness status	0, 1 vs 2	1.780 (1.339‐2.365)	<**0.0001**
Multivariate analysis			
N classification	N0 vs N1‐2	2.242 (1.384‐3.631)	**0.001**
Stemness status	0, 1 vs 2	1.883 (1.384‐2.562)	**<0.0001**

*P*‐values in bold indicates significance (*P* < 0.05).

Univariate analysis, Cox proportional hazards regression model

Multivariate analysis, Cox proportional hazards regression model.

Variables were adopted by univariate analysis.

### 3.5 ESCC CSCs’ confinement to CD47^+^ cells and the potential therapeutic significance of anti‐CD47

**Figure 4:**
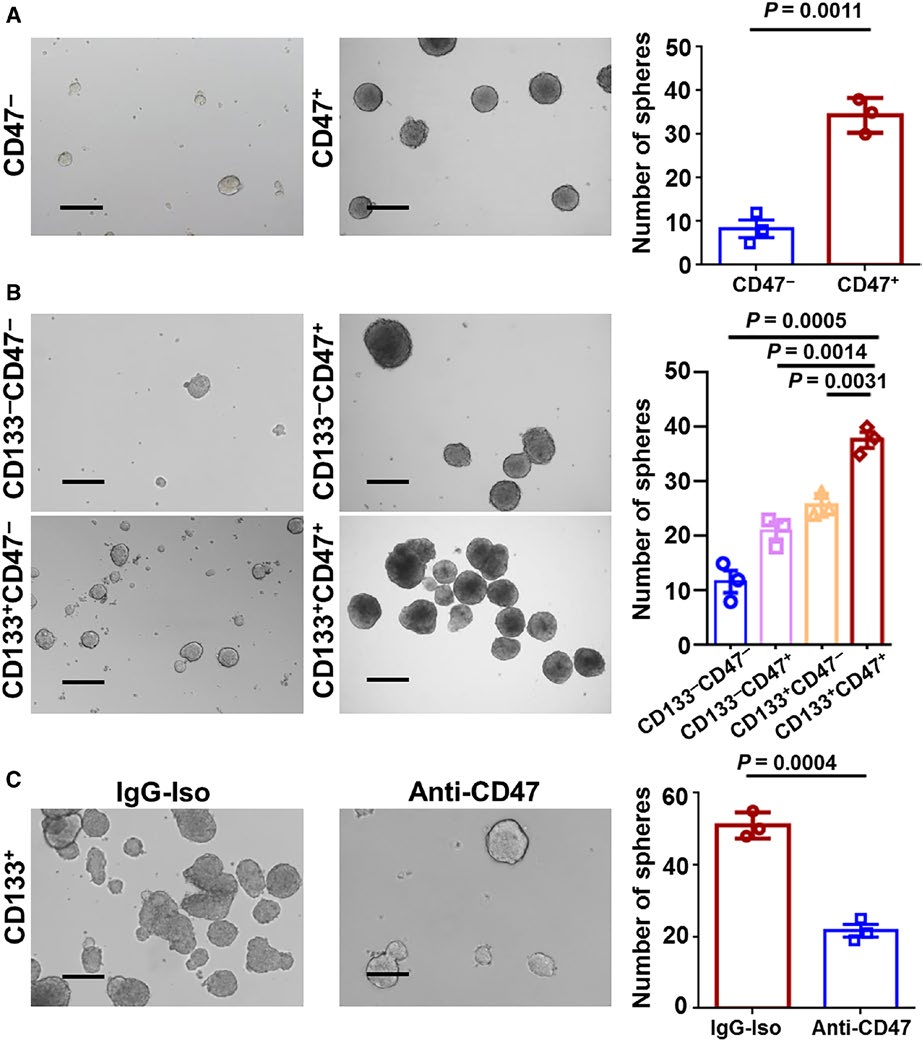
Anti‐CD47 treatment inhibits cancer stem cells (CSC) activity. A, Spheres in primary esophageal squamous cell carcinoma (ESCC) cells sorted for CD47. B, Sphere formation capability for cells FACSorted for CD47 and CD133. C, Sphere formation and quantification of cells after treatment with anti‐CD47 treatment, compared with IgG control treat cells. Scale bar = 500 μm. Data are expressed as mean ± SD (bars); Primary cells were obtained from ESCC patients and spheres were formed by culturing 3 × 10^3^ cells in three wells (n = 3). The number of spheres (>75 μm) was counted respectively. All experiments were performed in triplicate. Significance was determined using the Mann‐Whitney test

Given our data’s indication that CD47 is correlated with the differentiation of ESCC cells, we further aimed to explore whether CD47^+^CD133^+^ cancer cells were more stem‐like. We FACSorted primary ESCC cells from 6 patients for CD47 or CD133 and surveyed their self‐renewal capacity by sphere formation assay. We estimated the proportion of CD47^+^ cells in the CD133^+^ subpopulation, and as demonstrated in Figure S1A. Also, the most of CD133^+^ cells co‐expressed CD47 at an approximate percentage of 89%‐93% (Figure S1B). To further assess the CD47 characteristic in the ESCC CSCs, we first FACSorted primary ESCC cells for CD47^+^ and CD133^+^, then sorted four subpopulations based on their expressions intensity. We observed that more significantly larger spheres were formed by CD47^+^ cells than those by CD47^‐^ cells (Figure 4A). It indicated CD47^+^ cells are evidently enriched in CSCs and CD47^+ ^CD133^+^ cells distinctly possess with the strongest sphere formation capacity (Figure 4B). Furthermore, we confirmed that in contrast with the isotype‐matched IgG antibody treatment control cells, ESCCs cells treated with the human CD47 blocking antibody displayed effectively reduced sphere formation capacity in CD133^+^ cells (Figure 4C). Taken together, all these turn out that anti‐CD47 treatment had in fact eliminated the CSCs pool.

## 4 DISCUSSION

In the present study, we investigated the expression and prognostic potential of two important CSCs associated proteins, CD47 and CD133, and explored the potential therapeutic effect of anti‐CD47 treatment in ESCC. We found that they were both upregulated in primary ESCC patient samples, albeit to differing degrees. CD47 and CD133 correlated with poor prognosis, and the stemness index, a combination of CD47 and CD133, was an independent prognostic factor in ESCC for OS and PFS. Moreover, CD47^+^ cells were more “stem‐like” and anti‐CD47 treatment reduced the size and ratio of sphere formation in CD133^+^ ESCC cells.

Treatments with the inhibition of regulatory signals that are centrally relevant to CSCs capacity currently proved promising.^10^, ^20^ However, in preclinical studies, neutralizing antibodies against existing stemness markers have been proven insufficient for eradicating CSCs, likely due to the exceedingly heterogeneous genetic transcriptional response of cancer entailing larger populations of cells, which are resistant to single‐pathway targeting.^15^ Moreover, recent research has revealed that cancer stemness is partly defined by immune environmental cues.^21^ Thus, targeting CSCs based on broader immune therapeutics methods may be a more potent alternative for wiping out these thorny cells.

Growing evidence suggests that CD47 is required for the evasion of both innate^5^, ^22^ and adaptive^23^, ^24^ immune attacks and is verified to be preferentially overexpressed in CSCs, and we have pondered the possibility of CD47 being the sally port to ultimately eliminating CSCs. But CD47 seems not to be a perfect surrogate marker of CSCs, because of its high expression in majority cancer cells, including non‐CSCs, restricts the real enrichment CSCs numbers in CD47^+^ cells. Therefore, the combination of other CSCs markers would be required. Unlike CD47, which was strongly expressed in major neoplastic tissues, CD133 was slightly or moderately overexpressed in our data. A series of epitopes have been used as markers for identifying CSCs such as CD133, CD44, and CD90. More importantly, a recent study by Cheung et al^11^ identified CD133, but not CD90 or CD44, as a functional and targetable CSCs marker for ESCC. Therefore, we focused on assessing the stemness index prognosis power as determined by the combined expression of CD47 and CD133, utilized CD133 to make up for the lack of sensitivity of CD47 as a CSCs marker, and evaluated whether anti‐CD47 therapy could ably reduce the percentage of CSCs.

A number of previous studies exist on the identification and prognostic power of CD133 expression and other CSCs marker combinations. Allied expression of CD133 with CD15, CD44, aldehyde dehydrogenase or adenosine triphosphate‐binding cassette superfamily G member 2 has been suggested as a helpful tool in identifying putative CSCs and predicting the prognosis of patients.^25^, ^26^, ^27^, ^28^, ^29^ However, these combination strategies have yet to translate to expedient treatment. As the main objective in CSC research is to devise new means to effectively and selectively eliminate CSCs,^30^ our present work is focused on the combination of CD47 and CD133 as a potential predictor for ESCC patients, to further distinguish CSCs, and ultimately eliminating these malignant cells. Our data present first‐hand evidence for the correlation between concomitant high CD47‐CD133 expression and poor survival, as well as for CD47^+ ^CD133^+^ being an appropriate indicator to separate ESCC CSCs.

CD47 and CD133 were both proposed as potential predictors for CSCs. CD47, which has been identified as a “don’t eat me” signal to the immune system, is more highly expressed on CSCs of diverse origin, both in hematological and solid tumors. In our current study, CSCs from ESCC displayed a profound defect of sphere formation capacity by neutralizing CD47. What’s more, one proposed function of CD47 is to protect the CSCs from phagocytic clearance by macrophages. CD47^+ ^CD133^+^ CSCs may possess stronger self‐protection capability.^6^, ^9^, ^29^, ^30^ In ovarian, breast and pancreatic cancers, blockade CD47 signaling making CSCs more susceptible to be eliminated by macrophages.^15^, ^17^ All observations above suggest that CD47 can be a potential target for removing CSCs, thus preventing tumor relapse post‐conventional cytoreductive therapies. However, before CD47‐centered therapeutic approaches entering the clinical arena, the underlying mechanisms that regulate CD47 expression on CSCs must be addressed in the future. A Recent study has shown that, when exposed to hypoxic microenvironment, breast cancer cells increased their CD47 expression and in favor of a CSC‐like phenotype, which mediated by a direct binding of HIF‐1α to the CD47 promoter.[33] The Data from Shigemasa S et.al shows that CD47 is regulated by miR‐133a which probably function as a tumor suppressor.^7^ Our study provides preliminary proof that CD47 blockade may improve CSC eradicating strategy, while whether other novel and effective mechanisms exist merits further investigation.

## 5 CONCLUSIONS

We have uncovered that CD47 and CD133 are overexpressed in ESCC tissues, especially in poor differentiation tumors. CD133^+ ^CD47^+^ ESCC cells are found to possess the characteristics of CSCs. Inhibiting CD47 was an effective method of suppressing tumor stemness even at high levels in CD133^+^ cells. Although further mechanistic studies are still required for collation, our data presented—through combining CD47 and CD133 expression—the tumor stemness index as an independent prognostic factor for both OS and PFS. In the future, treatment and diagnosis based on the concomitant high CD47‐CD133 expression in ESCC may yet comprise a promising strategy.

## CONFLICTS OF INTEREST

No potential conflicts of interest were disclosed.

## ETHICAL APPROVAL

This investigation conformed strictly to the ethical guidelines of the Declaration of Helsinki, and the ethical approval was provided by the Research Ethics Committee of Sun Yat‐Sen University Cancer Center (Guangzhou, Guangdong province, China). We obtained surgical samples after obtaining written informed consent from all patients. All samples were coded, and the information was stored anonymously according to legal and ethical claim.

## Data Availability

These data have not been previously reported and is not under consideration for publication elsewhere.
