# Supplementary figures and images for: Combined prognostic value of the cancer stem cell markers CD47 and CD133 in esophageal squamous cell carcinoma
Source: Cancer Med. 2019 Feb 11;8(3):1315–25. doi: 10.1002/cam4.1894 (PMC6434369; doi:10.1002/cam4.1894)

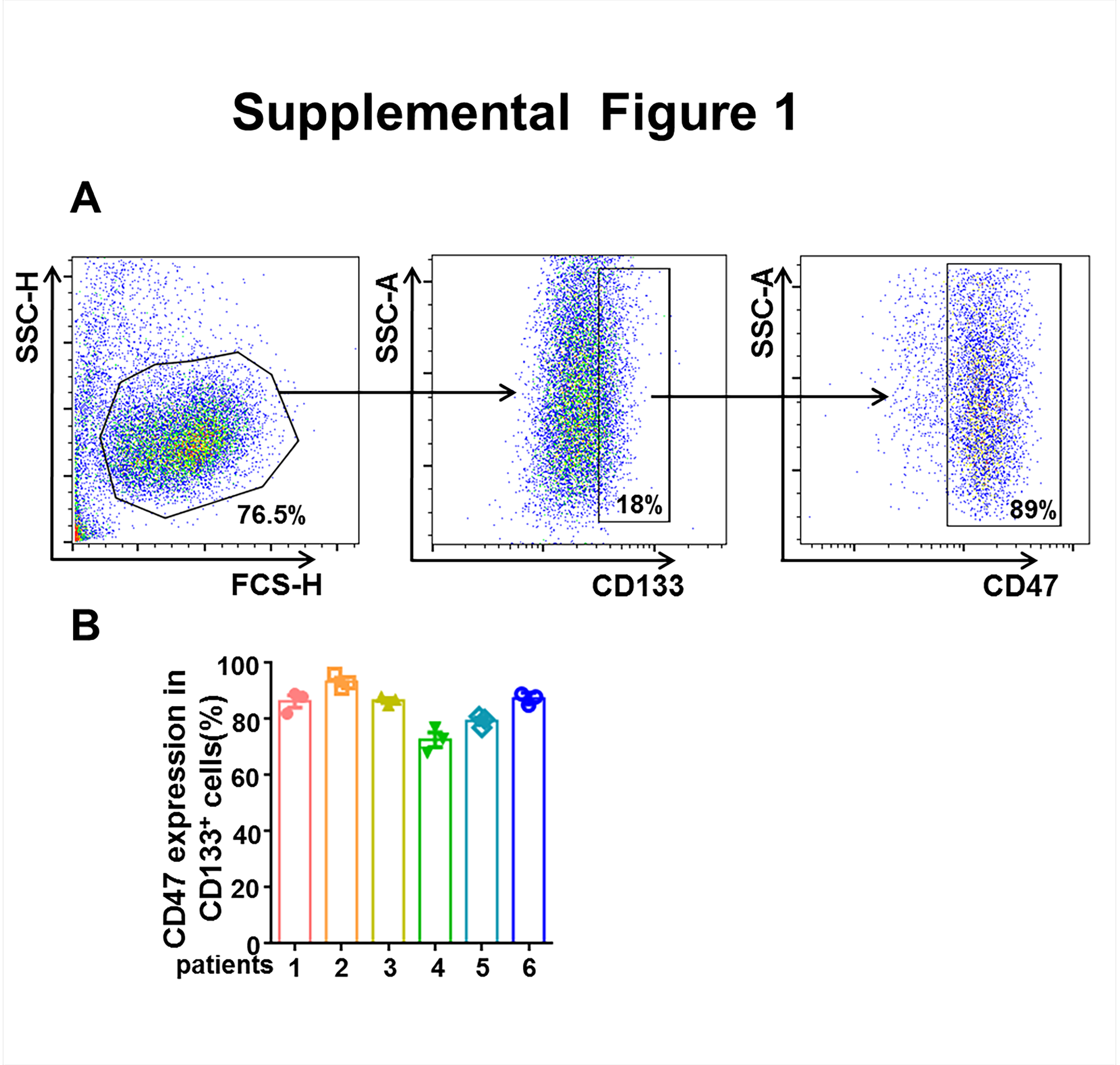

Supplement: Supplementary file 1 [file CAM4-8-1315-s001.tif]

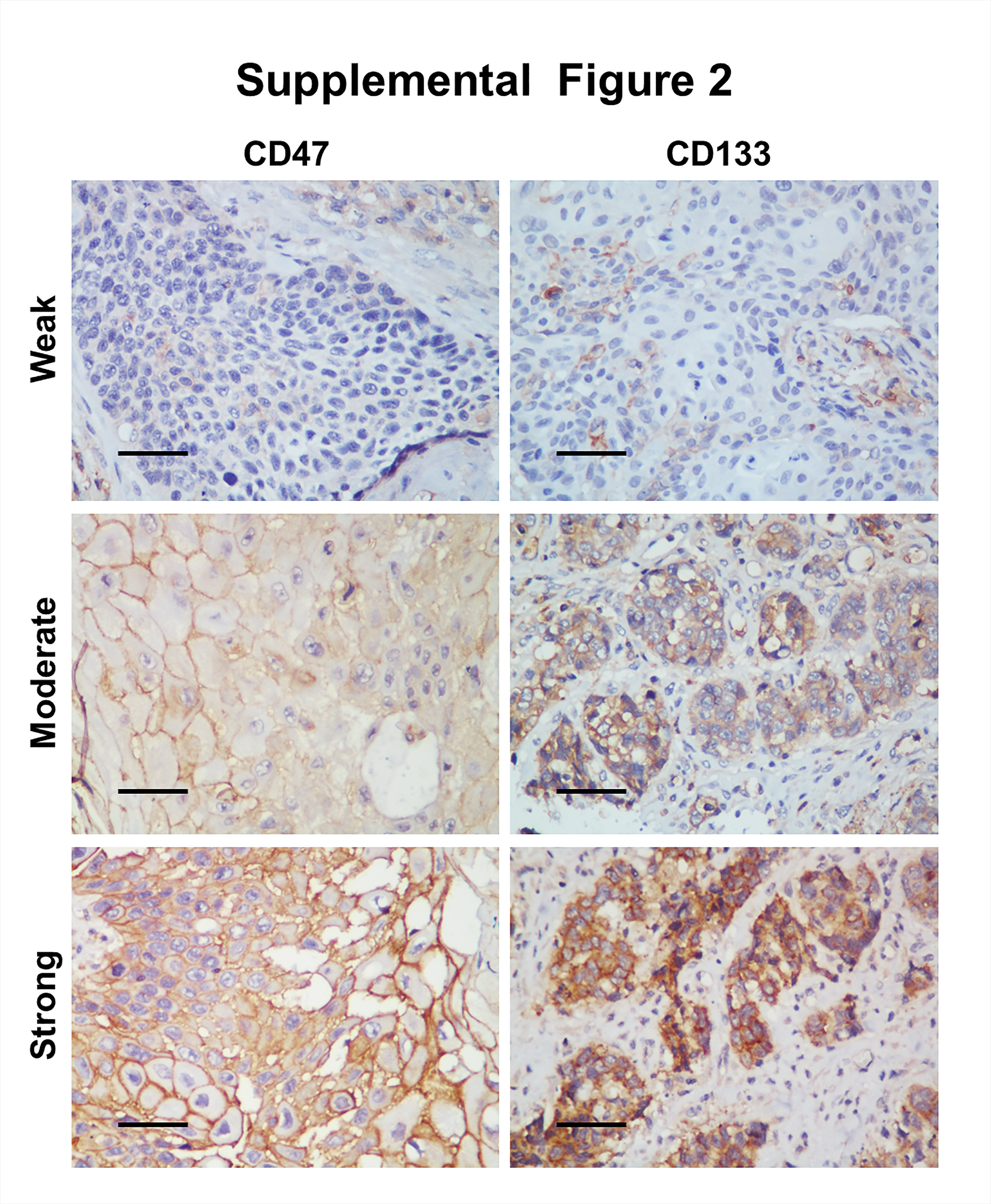

Supplement: Supplementary file 2 [file CAM4-8-1315-s002.tif]
